# Питание, ограниченное по времени, как новая стратегия терапии ожирения и коморбидных состояний

**DOI:** 10.14341/probl13078

**Published:** 2022-06-01

**Authors:** М. А. Берковская, О. Ю. Гурова, И. А. Хайкина, В. В. Фадеев

**Affiliations:** Первый Московский государственный медицинский университет имени И.М. Сеченова; Первый Московский государственный медицинский университет имени И.М. Сеченова; Первый Московский государственный медицинский университет имени И.М. Сеченова; Первый Московский государственный медицинский университет имени И.М. Сеченова

**Keywords:** ожирение, питание, ограниченное по времени, интервальное голодание

## Abstract

Настоящая рукопись представляет обзор современной литературы, посвященной изучению питания, ограниченного по времени (ПОВ), как инструмента лечения ожирения и коморбидных состояний. Поиск новых пищевых стратегий лечения ожирения, одной из которых является ПОВ, обусловлен слабой приверженностью пациентов гипокалорийным диетам в долгосрочной перспективе, а также имеющимися данными о важной роли десинхронизации приема пищи с естественными циркадианными ритмами в развитии и прогрессировании ожирения и кардиометаболических осложнений. В статье описываются основные механизмы, регулирующие циркадианные ритмы приема пищи и усвоения нутриентов, обосновывается важность соблюдения физиологического режима питания для поддержания метаболического здоровья. Основная часть обзора посвящена рассмотрению имеющихся на сегодняшний день исследований эффективности различных стратегий прерывистого ограничения энергии для снижения массы тела и коррекции метаболических показателей. Обсуждаются потенциальные механизмы влияния ПОВ на здоровье, в том числе опосредованные непреднамеренным снижением калорийности рациона и изменением пищевого поведения, различия в эффективности раннего и позднего ПОВ. Статья содержит подробное обсуждение потенциальных проблем и противоречий, связанных с внедрением питания, ограниченного по времени, в клиническую практику, а именно: ограниченность и противоречивость имеющихся клинических исследований, отсутствие данных о долгосрочной эффективности и безопасности, социальные и психологические ограничения, затрудняющие широкое использование ПОВ.

## Abstract (en)

The article provides a review of the current literature about time-restricted eating (TRE) as a new tool for the treatment of obesity and comorbid conditions. The search for new nutritional strategies in obesity, one of which is TRE, is due to the weak adherence of patients to hypocaloric diets in the long term, as well as the available data on the importance of ­desynchronization of food intake with natural circadian rhythms in the development and progression of obesity and cardio-­metabolic complications. The article describes the main mechanisms that regulate the circadian rhythms of food intake and nutrient absorption, substantiates the importance of adhering to a physiological diet for maintaining metabolic health. The main part of the review is devoted to reviewing the currently available researches on the effectiveness of various strategies of intermittent energy restriction for weight loss and the correction of metabolic parameters. Potential mechanisms of  the ­effect of TRE on health are discussed, including those mediated by an unintentional decrease in caloric intake and changes in eating behavior, and differences in the effectiveness of early and late TRE. The article contains a detailed discussion of the potential problems and contradictions associated with the use of time-restricted eating in clinical practice, namely: the limitations and inconsistencies of the available clinical trials, the lack of data on long-term efficacy and safety, social and psychological limitations that impede the widespread use of TRE.

## АКТУАЛЬНОСТЬ

Ожирение представляет собой заболевание, распространенность которого в настоящее время приобрела масштабы эпидемии и представляет реальную угрозу для общественного здоровья и здравоохранения. Частота ожирения прогрессивно нарастает в течение последних десятилетий: с 1975 по 2016 гг. доля населения Земли, имеющая ожирение, увеличилась с 5 до 13% и составила более 650 млн человек во всем мире [1]. Согласно прогнозам ВОЗ, к 2025 г. количество больных ожирением удвоится и будет составлять 30–50% населения экономически развитых стран [1]. Ожирение можно определить как хроническое гетерогенное системное заболевание, развивающееся в результате дисбаланса потребления и расхода энергии, характеризующееся избыточным накоплением жировой ткани и сопровождающееся высоким кардиометаболическим риском.

Жировая ткань, и в первую очередь висцеральный жир, помимо своей депонирующей функции играет важную роль в регуляции углеводного, жирового обмена, а также развитии кардиометаболических нарушений и осложнений, ассоциированных с ожирением. Жировая ткань представляет собой самостоятельный эндокринный орган, дисфункция которого, имеющая место при ожирении, обуславливает возникновение инсулинорезистентности, провоспалительного, проатерогенного и протромбогенного состояний, прогрессирование которых приводит к повышению риска развития сахарного диабета 2 типа (СД2) и атеросклеротических сердечно-сосудистых заболеваний (ССЗ). Учитывая широкое распространение ожирения и коморбидных состояний, трудно переоценить важность эффективного лечения ожирения, основной целью которого является, несомненно, снижение заболеваемости и смертности от ССЗ, СД2 и их осложнений.

Поскольку основными причинами ожирения являются избыточная калорийность пищи в совокупности со снижением энергозатрат, в том числе в результате недостаточной физической активности, основу лечения составляет сбалансированное антиатерогенное гипокалорийное питание. Умеренное ограничение калорийности (на 15–30% исходной) является наиболее распространенным подходом, рекомендуемым для борьбы с ожирением [2]. Не вызывает сомнений тот факт, что модификация образа жизни, включающая в себя здоровое питание, является неотъемлемой составляющей лечения ожирения. Однако низкая приверженность пациентов рекомендациям, направленным на изменение качества и калорийности рациона с помощью гипокалорийных диет, обуславливает низкую эффективность проводимых мероприятий [3][4]. Более того, этих стратегий сложно придерживаться в течение длительного времени, поэтому их эффективность в снижении кардиометаболических рисков у пациентов с ожирением ограничена.

В последние годы накапливаются данные о том, что помимо общей калорийности рациона важное значение в регуляции энергетического обмена, снижении и поддержании массы тела и улучшении кардиометаболических показателей имеет соблюдение режима питания. В современном мире нерегулярное питание, а также прием пищи в течение большей части суток (пищевое окно более 14–15 ч) являются обычным явлением [5][6] и могут быть связаны с ожирением, СД2, метаболическим синдромом и ССЗ [7][8]. Было показано, что более 50% людей едят в течение периода >15 ч каждый день и только около 10% взрослых обычно воздерживаются от еды в течение ≥12 ч в течение суток [6]. В американской популяции пациентов с метаболическим синдромом окно приема пищи, определяемое как временной интервал, в течение которого происходит 95% всех событий, связанных с приемом пищи, составляло примерно 15 ч [9]. Также было продемонстрировано, что нерегулярное время приема пищи отрицательно сказывается на кардиометаболическом здоровье [7]. Несоответствие между режимом питания и естественным циркадианным ритмом приводит к нарушению регуляции метаболизма и повышению кардиометаболических рисков [10–13].

Таким образом, поиск новых эффективных стратегий питания, направленных на лечение ожирения, метаболического синдрома и сопутствующих заболеваний, не утрачивает своей актуальности. Некоторые из диетических подходов, которые становятся все более популярными в последние годы, заслуживают особого внимания ввиду накапливающейся доказательной базы в отношении их позитивного влияния на массу тела и кардиометаболические показатели. В частности, интерес представляют стратегии, направленные не на тип, качество или количество продуктов, а в первую очередь на время приема пищи и продолжительность периода голодания: так называемое интервальное (периодическое) голодание и питание, ограниченное во времени (ПОВ). Данные подходы, нацеленные на нормализацию циркадианных ритмов за счет изменения времени и продолжительности ежедневного приема пищи, могут представлять собой многообещающую стратегию для пациентов с ожирением и метаболическим синдромом [14–18].

## ЦИРКАДИАННАЯ РЕГУЛЯЦИЯ ПРИЕМА ПИЩИ И МЕТАБОЛИЗМА

Циркадианные ритмы — это периодические колебания во внутренних биологических механизмах, которые определяют поведение и обменные процессы, такие как сон и бодрствование, работоспособность, гормональные сигналы, температура тела, потребление и усвоение питательных веществ [19]. Регуляция циркадианных ритмов осуществляется системой центральных и периферических механизмов. Центральный компонент регуляции включает супрахиазмальное ядро гипоталамуса (СХЯ), действующее как главный стимулятор синтеза и поддержания циркадианного ритма организма. Самым мощным регулятором циркадианных ритмов в рамках центрального контроля является свет. Сетчатка обнаруживает фотонные входы и преобразует их в СХЯ, что позволяет синхронизировать активность и метаболизм тканей и органов с циклами день/ночь. Эти циркадианные колебания генерируются белками, кодируемыми набором генов (например, Clock, Bmal1, Per и Cry), которые составляют петлю транскрипционно-трансляционной обратной связи [20]. Один цикл этой петли обратной связи занимает примерно 24 ч и является основой циркадианных ритмов у многих организмов, в том числе, у человека.

Помимо центральных часов, циркадианные ритмы подвержены влиянию периферических часов (таких как часы печени, поджелудочной железы, адипоцитов и т.д.), которые напрямую не стимулируются светом [19]. В отличие от СХЯ, периферические часы чрезвычайно чувствительны к циклу еда/голодание и могут быть десинхронизированы с центральными часами в результате изменений в режиме питания. Например, ограничение доступа к пище только световой фазой у мышей, когда эти ночные животные обычно спят, полностью изменяет фазу циркадианных часов в печени, желудке, кишечнике, сердце, поджелудочной железе и почках, не влияя на СХЯ [21]. Простая отсрочка приема пищи на 4 ч также приводит к сдвигу фазы циркадианных часов в печени мыши аналогичной продолжительности [22]. И наоборот, исследования показали, что ритмичного кормления достаточно для поддержания циркадианных ритмов часовых генов в периферических тканях во время постоянного света или темноты или после поражения СХЯ [23][24]. Эти данные предполагают, что сигналы цикла еда/голодание являются более мощными сигналами для периферических часов, чем цикл свет/темнота [25].

Циркадианные часы играют важную роль в регуляции жирового и углеводного обмена, определяя суточные колебания секреции гормонов. В то время как синтез таких гормонов, как мелатонин или кортизол, зависит в основном от ритмической активности СХЯ в ответ на свет/темноту, некоторые другие гормоны (грелин, инсулин) регулируются в основном циклами еда/ голодание [26].

Физиологически суточный цикл человека делится на фазу активности и фазу покоя. Эти две фазы зависят от мелатонина, «гормона сна», известного своей центральной ролью в регуляции циркадианных ритмов. Колебания мелатонина имеют особый паттерн, обычно используемый для определения дневных циклов. Секреция гормона начинается примерно в 22 ч, достигает пика в 3 ч ночи и, наконец, снижается к 10 ч утра. Она зависит от фотопериода и усиливается в ответ на низкую освещенность [20].

Около 4 ч утра в печени повышается секреция фактора роста фибробластов-21 (FGF-21). Она достигает максимума в 6 ч утра и снижается до минимума к полудню. FGF-21 является ключевым регулятором энергетического гомеостаза, способствуя активации АМФ-активируемой протеинкиназы (АМPK), что приводит к окислению жиров, увеличению гликолиза и ингибированию накопления субстратов [27][28].

Между 7 и 8 ч утра отмечается пик действия кортизола.. Этот гормон подготавливает организм к увеличению потребности в энергии, вызванной дневной активностью [29]. Производство кортизола связано с уменьшением секреции мелатонина, который обычно подавляет секрецию кортизола. Таким образом, цикл секреции кортизола противоположен циклу секреции мелатонина.

Гормон жировой ткани адипонектин начинает секретироваться около 10 ч утра и заканчивает примерно в 20 ч вечера, при этом пик секреции достигается в 12–14 ч [30][31]. Адипонектин является важным регулятором метаболизма глюкозы и липидов. Активируя АМРК, он улучшает гликолиз и окисление жирных кислот, а также снижает глюконеогенез в печени. Эти механизмы повышают утилизацию глюкозы и чувствительность к инсулину и предотвращают накопление жира [29][32].

Инсулин вырабатывается β-клетками островков Лангерганса поджелудочной железы и секретируется в ответ на повышенный уровень циркулирующих питательных веществ, особенно глюкозы. Инсулин оказывает анаболическое действие, стимулируя утилизацию глюкозы, липогенез и синтез белка периферическими тканями, такими как печень, скелетные мышцы и жировая ткань, а также ингибируя окисление жирных кислот. Секреция инсулина демонстрирует суточный ритм, определяющий накопление питательных веществ во время бодрствования/еды и последующую мобилизацию во время сна/голодания [30].

Секреция лептина достигает своего пика примерно в 19 ч вечера, а затем снижается до базального уровня в 2 ч ночи. Лептин определяет чувство насыщения, предотвращает прием пищи, увеличивает липолиз и ингибирует накопление жира [29].

Секреция грелина, синтезирующегося в фундальном отделе желудка, повышается в препрандиальный период и снижается вскоре после еды, что указывает на его роль в качестве возможного инициатора приема пищи. Несмотря на то что данный гормон регулируется в основном циклом еда/голод, он, по всей видимости, также подвержен суточной циркадианной регуляции, что было продемонстрировано в исследовании на здоровых добровольцах во время 24-часового голодания. Повышение и спонтанное снижение уровня грелина наблюдалось во время обычных приемов пищи. Корреляции грелина с уровнями гормона роста, инсулина или глюкозы в крови не наблюдалось, что указывало на сохранение центральной регуляции секреции [33].В другом исследовании на 120 здоровых добровольцах голодание в течение 3 дней устранило зависимость уровней грелина от еды, но суточные колебания его секреции сохранились и характеризовались более высокими уровнями в течение ночи с последующим снижением между 2:00-4:00 ч [34].

Таким образом, суточные ритмы секреции гормонов регулируют чередование катаболической и анаболической стадий, что важно для выполнения физиологических процессов в оптимальное время дня. Например, переваривание и метаболизм питательных веществ более эффективны, когда пища потребляется раньше в течение дня во время активной фазы, а не во время фазы покоя [35][36].

Исходя из описанного паттерна 24-часового колебания гормонов, регулирующих потребление и усвоение нутриентов, можно предположить, что оптимальным интервалом для потребления пищи может являться ранняя фаза активности. Действительно, между 8:00 и 16:00 вырабатываются FGF-21 и адипонектин, которые способствуют окислению жирных кислот, гликолизу и ингибируют накопление жира [28][37]. С другой стороны, логично предположить, что потребление пищи в более позднее время, в частности ночью, нежелательно, в том числе, исходя из максимальной секреции в это время лептина, который в норме вызывает чувство сытости [38].

Разумеется, данное предположение должно быть подтверждено дополнительными научными исследованиями, проведенными на животных и людях, однако уже сейчас накопленные научные данные свидетельствуют о том, что изменения такого естественного режима питания могут влиять на циркадианную систему и вызывать метаболические нарушения [20][39][40]. Так, показано, что поздний прием пищи (определяемый как ужин в течение 2 ч до отхода ко сну) связан с накоплением жировой ткани и повышенным риском ожирения [41][42]. Более того, люди, имеющие привычку поздно ужинать, склонны к потреблению больших порций, дополнительных порций и продуктов, обладающих высокой калорийностью; эти люди также имеют большую массу жировой ткани, инсулинорезистентность и повышенный риск ССЗ [43][44].

Точно так же, как прием пищи в неподходящее время способствует повышению метаболических рисков, пропуск еды, обычно потребляемой во время фазы активности, может иметь последствия для здоровья. В ряде исследований подчеркивается связь между пропуском завтрака и увеличением массы тела, сердечно-сосудистыми рисками и СД2, подтверждая тот факт, что употребление завтрака имеет важное значение для здорового режима питания [45]. В некоторых, однако не во всех исследованиях пропуск завтрака был ассоциирован с более высоким индексом массы тела и более высоким уровнем гликированного гемоглобина [46][47]. Также привычка не завтракать связана с повышением артериального давления и уровня холестерина липопротеинов низкой плотности (ЛПНП), что указывает на более высокий риск ССЗ [48][49].

В свете представленных фактов очевидно, что нарушение циркадианных ритмов, обусловленное изменениями режима питания, оказывает значительное влияние на метаболическое здоровье. Одним из возможных решений для лечения ожирения и снижения риска развития метаболических заболеваний в данном контексте может стать стратегия питания, ограниченного по времени, направленного на коррекцию образа жизни в соответствии с биологическими циркадианными ритмами. Данный подход в последние годы привлекает все большее внимание не только населения в целом, но и научного сообщества во всем мире, что служит основой для проведения исследований, направленных на изучение эффективности питания, ограниченного по времени, для снижения массы тела, коррекции кардиометаболических показателей и пищевого поведения пациентов с ожирением и метаболическим синдромом.

## ОПРЕДЕЛЕНИЕ СТРАТЕГИЙ ПРЕРЫВИСТОГО ОГРАНИЧЕНИЯ ЭНЕРГИИ

В настоящее время существует довольно много пищевых стратегий, подразумевающих периодические ограничения приема пищи или калорий (стратегии прерывистого ограничения энергии [50]). Основной проблемой в этой области является отсутствие общепринятой стандартизованной терминологии для описания различных парадигм прерывистого ограничения энергии. Так, ряд авторов используют термин прерывистое, или интервальное, голодание (intermittent fasting, ИГ) в качестве обобщающего термина для определения режимов питания, при которых люди проводят длительные периоды времени (например, 16–48 ч) с минимальным потреблением энергии или без нее, чередующиеся с периодами нормального приема пищи на регулярной основе [20][51][52]. М. Mattson и соавт. также разделяют стратегии краткосрочных частых периодов голодания и пищевые режимы с менее частыми, но более длительными периодами голодания, используя термин «периодическое голодание» (periodic fasting)) для обозначения режимов питания с периодами голодания продолжительностью от 2 до 21 и более дней [51]. Термин «питание, ограниченное по времени» (time-restricted eating, ПОВ) часто используется в качестве подкатегории ИГ для описания режима питания, при котором потребление пищи ограничено временным окном 8 ч или менее ежедневно в течение суток. Однако, по мнению других авторов, отнесение ПОВ к ИГ не совсем корректно, так как ПОВ принципиально отличается от полного дня/дней голодания или даже дня/дней, имитирующих голодание (обычно определяемого как день, в который люди потребляют до 25% дневной потребности в энергии) [50][53]. По словам С. Rynders и соавт., ПОВ — это скорее физиологический режим питания (потребление пищи днем, а не ночью), от которого люди отказались за последние несколько десятилетий [50]. Схемы наиболее распространенных режимов питания, подразумевающих прерывистое ограничение энергии, обобщены в таблице 1.

**Table:** Таблица 1. Наиболее распространенные режимы прерывистого ограничения энергииTable 1. Most Common Intermittent Power Limiting Modes

Протокол	Частота голодания	Длительность периода голодания	Описание
Питание, ограниченное по времени	Каждый день	14–18 ч	Прием пищи в течение 6–10 ч в течение суток. Окно приема пищи может быть определено протоколом или выбираться самостоятельно
Раннее питание, ограниченное по времени	Каждый день	18 ч	6-часовое окно приема пищи, включающее утренний завтрак и обед до 15–16:00
Режим В2	Каждый день	14 ч	2 приема пищи в день: завтрак между 6:00 и 10:00 и ланч между 12:00 и 14:00
Еженедельное суточное голодание	1 раз в неделю	24 ч	Только вода 1 день в неделю, питание ad libitum в остальные 6 дней
Протокол 5:2	2 раза в неделю	24 ч	Очень низкокалорийная диета (400–600 ккал/сут) 2 дня в неделю (в последовательные или непоследовательные дни), питание ad libitum в остальные 5 дней
Голодание через день	Через день	24 ч	Чередование дня голодания (0–25% суточной калорийности) с днем питания ad libitum
Комбинация ИГ с ограничением калорийности	Различная	24 ч	Пример протокола: гипокалорийная диета 6 дней в неделю (-30% суточной потребности), голодание (+120 ккал из сока) 1 день в неделю
Прерывистая очень низкокалорийная диета	Различная	24 ч	1.Очень низкокалорийное питание 1 раз в неделю. 2.Очень низкокалорийное питание в течение 5 последовательных дней 1 раз в 5 нед

## ФИЗИОЛОГИЧЕСКИЕ ЭФФЕКТЫ ПРЕРЫВИСТОГО ОГРАНИЧЕНИЯ ЭНЕРГИИ

Гипотеза об эффективности ПОВ и других пищевых стратегий, подразумевающих прерывистое ограничение энергии, в отношении лечения ожирения и ассоциированных с ним кардиометаболических нарушений основана на предположении о том, что введение регулируемых циклов приема пищи/голодания восстановит устойчивые циркадианные ритмы и улучшит метаболический гомеостаз.

С целью изучения влияния различных схем ПОВ на здоровье был проведен ряд клинических испытаний и исследований на животных моделях.

## Исследования на животных моделях ожирения и старения

В ряде исследований показано, что ограничение окна приема пищи фазой активности ограничивает набор массы тела и жировой массы, а также защищает ночных мышей и дневных мух от метаболических нарушений, индуцированных высокожировой диетой [54–61]. В том числе ПОВ оказывает противовоспалительный эффект [62], способствует усилению синтеза желчных кислот [54] и снижению уровня холестерина [55]. В исследованиях на животных моделях старения продемонстрировано, что ПОВ предотвращает вызванное возрастом и высокожировой диетой снижение сократительной функции сердца у мышей и мух [56][63] и восстанавливает потерю афферентной механочувствительности желудка и блуждающего нерва [64], а также ослабление циркадианных ритмов кишечного микробиома [65][66] и окисления жирных кислот [57][67]. Таким образом, эти данные позволяют предположить, что ПОВ обладает плейотропными метаболическими эффектами, направленными на защиту отхронических заболеваний у мышей и мух и, что важно, способно обратить вспять последствия ожирения [54] и старения [68]. Помимо схем ПОВ с ежедневным ограничением окна питания, положительные эффекты у мышей на высокожировой диете были также очевидны, когда ПОВ применялось 5 дней в неделю и доступ к пище был разрешен ad libitum в выходные дни [54][58].

На молекулярном уровне в указанных работах ПОВ увеличивало экспрессию AMPK и mTOR (mammalian target of rapamycin) [57][62] и никотинамидфосфорибозилтрансферазы (NAMPT) в печени мышей, получавших высокожировую диету [67], а также повышало экспрессию рибосомного белка фосфо-S6 (pS6) в скелетных мышцах во время активной фазы [54], предполагая повышенную активацию mTOR во время кормления. В печени ПОВ снижало активность пируваткарбоксилазы и глюкозо-6-фосфатазы и повышало активность глюкокиназы в активной фазе [54][57], что потенциально может вести к снижению глюконеогенеза печени и повышению утилизации глюкозы. ПОВ также способствовало снижению экспрессии генов синтазы жирных кислот, стеароил-Ко-десатуразы и элонгазы жирных кислот во время активной фазы и повышению экспрессии триглицерид-липазы в печени во время неактивной фазы, что было связано с уменьшением накопления липидов и повышенным гидролизом триглицеридов.

Важно отметить, что А. Chaix и соавт. исследовали эффекты ПОВ у мышей, у которых был дефицит циркадианных генов cry1 и cry2 на уровне всего тела или дефицит bmal1 и Rev-erbα/β только в печени. В этом исследовании ПОВ было эффективно для восстановления устойчивых ритмов в генах, участвующих в энергетическом метаболизме и использовании питательных веществ в печени при всех нокаутах, а также в путях передачи сигналов питательных веществ с более высокой функцией AMPKи mTOR. ПОВ также защищало нокаутированных животных от набора массы тела, индуцированного высокожировой диетой, нарушения толерантности к глюкозе, стеатоза печени и дислипидемии [67]. Это исследование доказывает, что соблюдение суточных ритмов в цикле голодания и кормления достаточно для поддержания метаболического гомеостаза, независимо от суточных часов [67].

## Исследования на людях

На сегодняшний день опубликовано немногим более 20 исследований различных режимов прерывистого ограничения энергии у людей, среди которых имеются как рандомизированные испытания, так и интервенционные исследования без контрольной группы (в том числе, перекрестные). Все имеющиеся на сегодняшний день исследования являются краткосрочными (от 4 дней до 16 нед) и достаточно малочисленными (от 8 до 116 участников) и включают в себя как людей с нормальной массой тела, так и пациентов с ожирением/избыточной массой тела (табл. 2). В большинстве исследований сообщается об умеренном снижении массы тела и жировой массы [9][14][69–74] и окружности талии [75]. ПОВ также способствовало снижению уровня триглицеридов и провоспалительных маркеров [72][73], артериального давления и проатерогенных липопротеинов [9][71]. В некоторых работах также было показано значимое улучшение контроля глюкозы [76][77], хотя данные результаты оказались непостоянными и не подтвердились другими авторами [9].

**Table:** Таблица 2. Наиболее значимые исследования прерывистого ограничения энергии у людей и их основные характеристикиTable 2. The most significant studies of intermittent energy restriction in humans and their main characteristics Сокращения: ПОВ — питание, ограниченное по времени; ПО — пищевое окно; AL — ad libitum; ИМТ — индекс массы тела; CGM — непрерывное мониторирование глюкозы; HbA1c — гликированный гемоглобин; Ж — женщины; М — мужчины; ПГТТ— пероральный глюкозотолерантный тест; СД2 — сахарный диабет 2 типа; ОТ — окружность талии.

Исследование и дизайн	Критерии включения	Участники	Длительность исследования и пищевого окна	Время приема пищи во время ПОВ	Основной результат
М. Wilkinson et al. [9]Пилотное неконтролируемое исследование	Метаболический синдром (≥3 компонентов)ПО≥14 ч	n=19 (6 женщин, 13 мужчин)Ожирение с метаболическим синдромомПО 15,1±1,1 чВозраст 59±11,1 года	12 недПО 10 ч	Самостоятельно выбранный интервал приема пищи	Изменение среднего уровня глюкозы в крови (CGM)
L. Chow et al. [14]Рандомизированное контролируемое исследование	Возраст 18–65 летИМТ≥25 кг/м2ПО≥14 ч	n=20 (17 Ж, 3 М)Избыточный вес/ожирениеПО 15,2±0,7 чПОВ: n=11 (9 Ж, 2 М)Возраст 46,5±12,4 гКонтроль:n=9 (8 Ж, 1 М)Возраст 44,2±12,3 года	12 недПО 8 ч	ПОВ: самостоятельно выбранный интервал приема пищиКонтроль: AL	Изменение массы тела
E. Sutton et al. [69]Перекрестное исследование	Мужчины;Возраст 35–70 лет;ИМТ 20–39 кг/м2Преддиабет (по данным HbA1c и ПГТТ)	n=8 (M)Избыточный вес/ожирение и предиабетВозраст 56±9 лет	5 нед в каждом режимеПО 6 или 12 ч	Ранее ПОВ: с 8:00 до 14:00(ужин до 15:00) Контроль: с 8:00 ;до 20:00	Изменения толерантности к глюкозе, постпрандиального инсулина и чувствительности к инсулину
S. Anton et al. [70]Пилотное неконтролируемое исследование	Возраст ≥65 летИМТ 25–40 кг/м2Малоподвижный образ жизни	n=10 (6 Ж, 4 М)Избыточный вес/ожирениеВозраст ≥65 лет	4 недПО 8 ч	Самостоятельно выбранный интервал приема пищи	Осуществимость и безопасность ПОВ у пожилых людей с избыточным весом
K. Gabel et al. [71]Пилотное неконтролируемое исследование	Возраст 25–65 летИМТ 30–45 кг/м2Малоподвижный образ жизни	n=46 (41 Ж, 5 М)ОжирениеПОВ: n=23 (20 Ж, 3 М)Возраст 50±2 годаКонтроль: n=23 (21 Ж, 2 М)Возраст 48±2 года	12 недПО 8 ч	ПОВ: 10:00–18:00Контроль: AL	Изменение массы тела
T. Moro et al. [73]Рандомизированное контролируемое исследование	Тренирующиеся мужчины (тренировки 3–5 дней в неделю с опытом сплит-тренировок не менее 3 лет)	n=17 (возраст 29,94±4,07 лет), контрольная группа n=17 (возраст 28,47±3,47 года)	12 недПО 8 ч	ПОВ: 13:00–20:00Контроль: 8:00–20:00Протоколы изоэнергетической диеты	Снижение жировой массы, тестостерона и ИФР-1 в группе ПОВ
K. Stote et al. [74]Рандомизированное перекрестное исследование	Здоровые взрослые с нормальной массой тела	n=15 (10 Ж, 5 M)Возраст 45±0,7 года	8 нед в каждом режимеПО 4 ч	ПОВ: 1 прием пищи в 17:00-21:00 Контроль: 3 приема пищи Протоколы изоэнергетической диеты	Уменьшение жировой массы, усиление чувства голода
D. Kesztyüs et al. [75]Пилотное неконтролируемое исследование	Один или несколько компонентов метаболического синдрома, включая СД2 без инсулинотерапии	n=40 (31 Ж, 9 М)Абдоминальное ожирениеВозраст 49,1±12,4 года	12 недПО 8–9 ч	Самостоятельно выбранный интервал приема пищи	Приверженность вмешательству (доля дней с голоданием ≥15 ч)
А. Hutchison et al. [76]Перекрестное исследование	Возраст 30–70 летОТ≥102 смВысокий риск СД2	n=15 (М)Ожирение (абдоминальное ожирение)Возраст 55±3 года	7 дней в каждом режимеПО 9 ч	Раннее ПОВ: с 8:00 до 17:00 Позднее ПОВ: с 12:00 до 21:00	Реакция глюкозы на смешанную пищу у мужчин с риском развития СД2
Н. Jamshed et al. [77], Е. Ravussin et al. [93]Перекрестное исследование	Здоровые взрослыеВозраст 20–45 летИМТ 25–35 кг/м2	n=11 (4 Ж, 7 М)Избыточный вес/ожирениеВозраст 32±7 лет	4 дняПО 6 или 12 ч	Ранее ПОВ: с 8:00 до 14:00Контроль: с 8:00 до 20:003-разовое питание, предоставляется и распределяется по группам	Изменение расхода энергии
М. Gasmi et al. [81]Нерандомизированное контролируемое исследование	Здоровые взрослые мужчины	n=10 Возраст 26,90±1,97 года, n=10 Возраст 51,60±5,87 года	12 недПО 12 ч	ПОВ: 12-часовое окно приема пищиКонтроль: AL	Снижение маркеров воспаления
G. Tinsley et al. [82]Рандомизированное контролируемое исследование	Здоровые активные мужчины	n=10 Возраст 22,9±4,1 года	8 недПО 4 ч	ПОВ: потреблении всех калорий в течение 4-часового окна в течение 4 дней в неделю без ограничения продуктов и калорий	Снижение потребления калорий без влияния на состав тела
R. Singh et al. [84]Рандомизированное перекрестное исследование	Здоровые взрослые с нормальной массой тела	n=22 (2 Ж, 20 M)Возраст 30,9±8,95 года	4 нед 1 прием пищи в день (утро vs вечер)	Основная часть рациона (около 2000 ккал) утром или после 20:00	Изменение массы тела, уровня гликированного гемоглобина
R. Antoni et al. [85]Рандомизированное контролируемое исследование	Здоровые взрослыеИМТ 20–39 кг/м 2	n=13 (12 Ж, 1 М)ПОВ: n=7 (6 Ж, 1 М)Возраст 47±3 годаКонтроль: n=6 (Ж)Возраст 45±4 года	10 недПО 8 ч 37 мин±22 мин	ПОВ: завтрак откладывается, а ужин переносится на 1,5 чКонтроль: AL	Возможности ПОВ для сужения окна приема пищи
C. Martens et al. [86]Рандомизированное контролируемое исследование	Здоровые взрослые без ожирения	n=22 (12 Ж, 10 M)Возраст 67±1 лет	6 нед ПО 8 ч	ПОВ: 8-часовое окно приема пищиКонтроль: AL	Безопасность и переносимость ПОВ
M. McAllister et al. [87]Рандомизированное контролируемое исследование	Физически активные студенты колледжа	n=22 (22 M);Возраст 22±2.5 лет	4 нед ПО 8 ч	ПОВ: питание 16:8; Протокол изоэнергетической диеты vs контроль – ПОВ без ограничения калорий	Снижение жировой массы, АД, повышение адипонектина, ХС-ЛПВП
S. Cienfuegos et al. [94]Рандомизированное контролируемое исследование	Возраст 18–65 годаИМТ 30–49,9 кг/м2Малоподвижный или умеренно активный образ жизни	n=58 (53 Ж, 5 М)Ожирение4-часовой ПОВ:n=19 (17 Ж, 2 M)Возраст 47±2 года6-часовой ПОВ:n=20 (19 Ж, 1 M)Возраст 47±3 годаКонтрольная группа:n=19 (17 Ж, 2 М)Возраст 45±2 года	8 недПО 4 или 6 ч	4-часовой ПОВ: 15:00–19:006-часовой ПОВ: 13:00–19:00Контроль: AL	Изменение массы тела
D. Lowe [95]Рандомизированное контролируемое исследование	Возраст 18–64 годаИМТ 27–43 кг/м2	n=116 (46 Ж, 70 М)Избыточная масса тела/ожирениеВозраст 46,5±10,5 летОчная группа ПОВ:n=25 (12 Ж, 13 М)Возраст 43,3±11,8 года	12 недПО 8 ч	ПОВ: с 12:00 до 20:00Постоянное время приема пищи: />3 структурированных приема пищи в день	Изменение массы тела
Е. Parr et al. [99]Рандомизированное контролируемое перекрестное исследование	МужчиныВозраст 30–45 летИМТ 27–35 кг/м2Малоподвижный образ жизни	n=11 (M)Избыточный вес/ожирениеВозраст 38±5 летГруппа 1: n=6Группа 2: n=5	5 днейПО 8 ч	ПОВ: 10:00–18:00Контроль: 7:00–22:00Протоколы изоэнергетической диеты	Циркадианные профили глюкозы и инсулина в крови

Несмотря на впечатляющие результаты, продемонстрированные некоторыми авторами представленных исследований, метаанализ библиотеки Coсhrane, опубликованный в 2021 г., не поддержал преимуществ ИГ (питания, ограниченного повремени), по сравнению с гипокалорийной диетой, в лечении ожирения и коморбидных состояний [78]. В данный обзор было включено 18 рандомизированных контролируемых испытаний (1125 участников), сравнивающих ИГ с питанием ad libitum (прием пищи в любое время без определенного ограничения калорийности) или с постоянным ограничением калорий (гипокалорийная диета). Включались исследования с участием взрослых людей (старше 18 лет) с кардиометаболическими факторами риска и без них. Режимы питания включали в себя голодание через день, модифицированное голодание через день, периодическое голодание и питание, ограниченное по времени. В семи исследованиях сравнивали ИГ с питанием ad libitum, в восьми — с гипокалорийной диетой и в трех — с питанием ad libitum и гипокалорийной диетой. Авторы регистрировали результаты при краткосрочном (≤3 мес) и среднесрочном (от 3 до 12 мес) наблюдении.

По данным проведенного анализа отмечалось снижение массы тела на фоне ИГ по сравнению с питанием ad libitum в краткосрочной перспективе (медиана -2,88 кг, 95% ДИ от -3,96 до -1,80, 224 участника, 7 исследований; доказательства с низкой степенью достоверности). В отношении снижения массы тела на фоне ИГ по сравнению с гипокалорийной диетой в краткосрочной перспективе значимость результатов была пограничной (медиана -0,88 кг, 95% ДИ -1,76 до 0,00, 719 участников, 10 исследований, доказательства с очень низкой степенью достоверности) и отсутствовала в среднесрочной (медиана -0,56 кг, 95% ДИ от -1,68 до 0,56, 279 участников, 4 исследования, доказательства с низкой степенью достоверности).

Значимых изменений в уровне глюкозы на фоне ИГ по сравнению с питанием ad libitum и гипокалорийной диетой выявлено не было: по сравнению с питанием ad libitum в краткосрочной перспективе медиана -0,03 ммоль/л, 95% ДИ -0,26–0,19; 95 участников, 3 исследования, очень низкая достоверность доказательств; по сравнению с гипокалорийной диетой в краткосрочной перспективе: медиана -0,02 ммоль/л, 95% ДИ -0,16–0,12, 582 участника, 9 исследований, очень низкая уверенность; среднесрочная перспектива: медиана 0,01, 95% ДИ от -0,10 до 0,11, 279 участников, 4 исследования, доказательства с низкой достоверностью.

Среди возможных объяснений непостоянства результатов, полученных в исследованиях режимов прерывистого ограничения энергии на людях, может предположить различие эффектов, оказываемых ранним и поздним питанием, ограниченным повремени [20], а также неоднородность результатов, полученных в исследованиях на лицах с или без избыточной массы тела/ожирения [53].

Так, в метаанализе S. Moon и соавт. [53] показано, что в подгруппе исследований, включавших участников с метаболическими нарушениями [70][76][79–81], отмечалось достоверное снижение массы тела (медиана -3,19; 95% ДИ от -4,62 до -1,77), вто время как в подгруппе исследований с участием здоровых добровольцев [74][81–85] значимого изменения массы тела продемонстрировано не было (медиана 0,17; 95% ДИ от -0,81 до 1,15). При анализе чувствительности одно исследование [80] показало значительное влияние на результат. Когда оно был удалено, статистическая значимость исчезла (медиана -0,01; 95% ДИ от -0,95 до 0,93). Схожая ситуация наблюдалась при анализе влияний ПОВ на показатели углеводного обмена. ПОВ значимо снижало концентрацию глюкозы натощак в работах, включавших участников с метаболическими нарушениями [9][70][71][77][80] (медиана -2,29; 95% ДИ от -4,29 до -0,19), однако в исследованиях со здоровыми участниками [73][82][85–87] значимых изменений гликемии натощак на фоне ПОВ продемонстрировано не было, несмотря на тенденцию к снижению данного показателя (медиана -3,65; 95% ДИ от -7,65 до 0,35).

Обсуждение метаболических эффектов ПОВ требует также рассмотрения вопроса о различном влиянии раннего и позднего ограниченного по времени питания. В ряде работ показано, что питание, ограниченное по времени ранней фазой активности, может быть более благоприятным с метаболической точки зрения. Так, результаты наблюдательных и эпидемиологических исследований показывают, что люди, пропускающие завтрак, чаще имеют избыточную массу тела и ожирение, гипергликемию и СД2, по сравнению с людьми, которые регулярно завтракают [88]. В то же время другие обсервационные исследования показали, что пропуск завтрака без позднего приема пищи не связан с ожирением, неоптимальным гликемическим контролем или ухудшением метаболического здоровья [89][90][91]. В исследовании D. Jakubowicz и соавт. тучные женщины были рандомизированы для получения высококалорийного завтрака по сравнению с высококалорийным ужином, при этом в группе, получавшей высококалорийный завтрак, отмечалось большее снижение массы тела, окружности талии, глюкозы и инсулина натощак [92]. В целом, опираясь на имеющиеся на сегодняшний день данные, можно предположить, что раннее ограниченное по времени питание, когда прием пищи происходит с 8:00 до 18:00, способствует снижению массы тела и аппетита у людей с избыточной массой тела и ожирением [69][71][93][94], уровня артериального давления и холестерина ЛПНП [79][81]; а у пациентов с предиабетом и СД2 ассоциировано с более низким уровнем инсулина и улучшением чувствительности к нему [69][75]. Вместе эти исследования показывают, что прием пищи в начале дня является оптимальным для контроля массы тела и улучшения гликемического профиля в изокалорийных условиях.

Эти результаты могут указывать на важность согласования приема пищи с циркадианными ритмами для улучшения кардио-метаболических показателей. Действительно, в нескольких исследованиях использовалось позднее ограниченное повремени питание, когда испытуемым разрешалось есть до вечера, что сопровождалось снижением положительных эффектов. Результаты данных работ оказались непостоянны: S. Cienfuegos с соавт. продемонстрировали снижение массы тела [94], тогда как в других исследованиях значимого изменения массы тела или общей жировой массы [95][96] зарегистрировано не было. Кроме того, отсутствовало значимое влияние позднего ПОВ на уровень глюкозы натощак, гликированного гемоглобина, триглицеридов, общего холестерина, артериального давления, уровни ЛПНП или ЛПВП [94][96]. Эти результаты могут отражать важность соответствия окна приема пищи и циркадианных ритмов для оптимизации эффектов ограниченного по времени питания и, следовательно, для улучшения здоровья.

В контексте вышесказанного было бы интересно сравнить эффекты раннего и позднего ограниченного по времени питания, чтобы лучше понять важность конкретизации ежедневного окна приема пищи для достижения благоприятного результата этой стратегии. На данный момент известно только одно исследование, сравнивающее эффекты раннего (с 8:00 до 17:00) и позднего (с 12 до 21:00) ограниченного по времени питания на показатели углеводного обмена [76]. По данным авторов, питание с ограничением по времени улучшает гликемический ответ независимо от времени приема пищи (позднего или раннего), хотя в группе раннего питания с ограничением по времени средний уровень глюкозы натощак был ниже, чем в группе позднего [76]. Может показаться, что результаты данного исследования несколько дискредитируют идею о важности раннего приема пищи при использовании стратегии ПОВ. Однако это исследование имеет несколько ограничений: в него были включены только мужчины, поэтому эти результаты не могут быть экстраполированы на женщин; количество участников было невелико (всего пятнадцать); группы раннего и позднего питания с ограничением по времени были протестированы в течение короткого периода времени (одна неделя).

Таким образом, чтобы сделать вывод о наиболее эффективной стратегии питания, особенно в отношении различий между ранним и поздним ограниченным по времени питанием, требуются более крупные и продолжительные рандомизированные клинические испытания. Кроме того, существенным ограничением имеющихся на данный момент исследований, препятствующим возможности делать окончательные заключения, является отсутствие стандартизации протокола питания, ограниченного по времени.

## ПИТАНИЕ, ОГРАНИЧЕННОЕ ПО ВРЕМЕНИ, И ЭНЕРГОЕМКОСТЬ РАЦИОНА

Исследования, опубликованные на сегодняшний день, не позволяют определить, улучшает ли питание, ограниченное по времени, показатели здоровья, независимо от изменений энергоемкости рациона. Некоторые авторы предполагают наличие независимых от ограничения калорийности положительных метаболических эффектов ПОВ (снижение уровня системного воспаления и окислительного стресса) [54][67]. Тем не менее, воздействие прерывистого ограничения энергии на уровень воспаления и окислительного стресса у людей изучено минимально и неясно, оказывает ли данная стратегия положительное влияние на данные патологические процессы у человека [97].

Следует также отметить, что точную калорийность потребляемой пищи трудно измерить, кроме того, в ряде работ показано снижение потребления калорий у мышей на высокожировой диете при соблюдении ограниченного по времени кормления [55][59] и заметное первоначальное снижение массы тела в ответ на ПОВ [59][64]. Снижение потребления калорий на ~9–20% было зарегистрировано в исследованиях ПОВ с участием людей с избыточной массой тела или ожирением, включая пациентов с метаболическим синдромом, несмотря на отсутствие рекомендаций по изменению количества и качества продуктов во время вмешательства [9][14][71][94]. У взрослых с ожирением без явных метаболических осложнений потребление энергии без подсчета калорий сократилось на 20%, отчасти из-за уменьшения потребления алкогольных напитков и ночных перекусов [72]. Важно отметить, что 4-часовое и 6-часовое ПОВ приводили к сопоставимому снижению потребления энергии (на ~550 ккал/день) без целенаправленного подсчета калорий у взрослых с ожирением, по сравнению с контрольной группой [94]. В некоторых исследованиях на фоне ПОВ наблюдалось непреднамеренное снижение потребления энергии одновременно с усеченным окном приема пищи, что свидетельствует о том, что реализация данной стратегии питания без снижения калорийности труднодостижима для людей из-за спонтанного ограничения энергии в течение периода исследования [9][71].

Снижение потребления энергии может объяснить некоторые положительные эффекты ПОВ на массу тела и метаболические исходы. Таким образом, по крайней мере некоторые метаболические преимущества ПОВ могут быть опосредованы ограничением калорийности и снижением массы тела. Тем не менее исследование на мышах показало, что ПОВ улучшает толерантность к глюкозе и снижает индекс инсулинорезистентности HOMA-IR у крыс после диеты с высоким содержанием жиров и сахара без какой-либо потери массы тела [61]. Недавнее исследование на людях также подтвердило мнение о том, что ПОВ имеет метаболические преимущества независимо от изменений массы тела [69]. Возможные механизмы данного влияния на являются предметом дальнейшего изучения.

## ПИТАНИЕ, ОГРАНИЧЕННОЕ ПО ВРЕМЕНИ, И ПИЩЕВОЕ ПОВЕДЕНИЕ

Важным вопросом, возникающим при рассмотрении прерывистого ограничения энергии в качестве долгосрочной стратегии для снижения массы тела и улучшения метаболического здоровья, является потенциальная проблема изменения пищевого поведения в результате соблюдения данного режима питания. Согласно результатам некоторых исследований, ИГ может оказывать влияние на пищевое поведение и эмоциональное состояние, в т.ч. на частоту депрессии и компульсивного переедания. Компульсивное переедание — это употребление аномально больших объемов пищи за короткий период времени. Известно, что компульсивным перееданием страдают 2,8 млн американцев, причем наибольшую распространенность оно имеет среди людей с ожирением и тех, кто стремится похудеть [97]. Примерно 50% лиц, имеющих признаки ограничительного пищевого поведения, отмечают эпизоды компульсивного переедания.

К. Hoddy и соавт. обнаружили, что 8 нед голодания через день (alternate-day fasting) у 59 лиц с ожирением привели к уменьшению депрессии и переедания (p<0,01) [98]. Хотя снижение депрессии и переедания было статистически значимым, оно не казалось клинически значимым, поскольку абсолютные изменения были минимальными. В ряде других исследований также продемонстрировано положительное влияние ПОВ на психологические и поведенческие параметры испытуемых: улучшение сна, повышение уровня энергии, качества жизни и благополучия, снижение субъективного чувства голода перед сном [9][69–71][76][77][93][99]. Раннее 6-часовое ПОВ привело к снижению аппетита и, несмотря на более длительную продолжительность ежедневного голодания, к уменьшению чувства голода вечером, что в совокупности может способствовать снижению массы тела [69][93]. Так, в исследовании Е  Ravussin и соавт. раннее ПОВ способствовало снижению массы тела, в первую очередь за счет снижения аппетита и калорийности суточного рациона, а не за счет увеличения расхода энергии [93]. Поэтому согласование приема пищи с циркадианными ритмами может быть хорошей стратегией для снижения аппетита и похудания.

Несмотря на краткосрочные психоэмоциональные преимущества, продемонстрированные в исследованиях схем прерывистого ограничения энергии, длительное использование данных пищевых стратегий в настоящее время не изучено в плане их влияния на пищевое поведение. Например, длительные периоды голодания могут завершаться употреблением больших порций нездоровой пищи (компенсаторной гиперфагической реакцией), в связи с чем возникает закономерный вопрос, насколько возможно сохранить преимущества ИГ/ограниченного по времени питания для снижения массы тела в течение длительного времени [97]. В целом, прежде чем рекомендовать различные схемы прерывистого ограничения энергии в качестве долгосрочной стратегии борьбы с ожирением и коморбидными состояниями, необходимы более крупные и длительные контролируемые испытания, оценивающие долгосрочные взаимосвязи между ПОВ и изменениями пищевого поведения.

## БАРЬЕРЫ ДЛЯ ИСПОЛЬЗОВАНИЯ ПИТАНИЯ, ОГРАНИЧЕННОГО ПО ВРЕМЕНИ

Несмотря на рассмотренные потенциальные преимущества для здоровья, широкие рекомендации ПОВ в настоящее время представляются не вполне обоснованными, поскольку клинические испытания данного подхода на людях достаточно ограничены, краткосрочны, разнородны по исследуемым популяциям и зачастую дают противоречивые результаты. Кроме того, ПОВ имеет целый ряд недостатков и ограничений, которые могут сдерживать его внедрение. Одним из наиболее значимых лимитирующих моментов является дневное окно приема пищи до 16–18 ч, поскольку его трудно соблюдать при сохранении семейной, социальной или рабочей жизни. Действительно, работа в ночную смену затрудняет соблюдение ИГ из-за его десинхронизации с графиком работы [100]. Семейный образ жизни также может быть тормозом для ограниченного по времени питания, потому что время семейного приема пищи обычно соответствует общепринятым моделям питания современного общества (завтрак, обед и ужин). Ужин зачастую является самым важным приемом пищи в семейной обстановке. Он облегчает и укрепляет общение между членами семьи и способствует социально-эмоциональному развитию и психическому здоровью [101]. Еда является частью общественной и культурной жизни, которая способствует социальным контактам путем встреч с друзьями, коллегами или родственниками. Товарищество, определяемое актом приема пищи с другими видами совместной деятельности, предоставляет возможности для социальной интеграции, поддержки и общения [102]. Следовательно, питание, отличное от привычного для окружения, может стать препятствием для социального и семейного развития, способствовать возникновению изоляции, одиночества или депрессии [103][104].

Еще одним ограничением ПОВ является отсутствие стандартизации его протокола. До сих пор не существует единого мнения об идеальном времени для приема пищи/голодания или оптимальной продолжительности каждого из окон. В исследованиях используется разная продолжительность периода питания, от 8 до 10 ч, и рекомендуются различные временные интервалы для интермиттирующего ограничения калорий [20]. Хотя нет никаких доказательств серьезных побочных эффектов ПОВ в том случае, если оно синхронизировано с циркадианными часами [9], некоторые авторы отмечают важность уточнения времени при выборе окна приема пищи. По имеющимся на сегодняшний день данным, ПОВ улучшает метаболическое здоровье в основном тогда, когда время приема пищи совпадает с ранней фазой активности. И наоборот, ИГ, при котором окно приема пищи приходится на вечернее время, может быть связано с увеличением массы тела, жировой массы и гликемии [85][104].

Таким образом, внедрение ПОВ требует строгого контроля, сопряжено с трудностями соблюдения временных интервалов приема пищи, что может мешать нормальной социальной и семейной жизни, не совпадать с рабочим графиком. Кроме того, учитывая ограниченность имеющихся на сегодняшний день научных данных в отношении оптимального режима ПОВ для людей и его положительных эффектов на метаболическое и психическое здоровье, широкая рекомендация данной стратегии питания для борьбы с ожирением и коморбидными состояниями представляется преждевременной.

## ЗАКЛЮЧЕНИЕ

Растущая пандемия ожирения и сопутствующих ему сердечно-сосудистых заболеваний диктует необходимость поиска новых современных, эффективных и безопасных инструментов снижения массы тела, в т.ч. пищевых стратегий. В последние годы все больше внимания уделяется режимам интермиттирующего ограничения энергии (ПОВ, ИГ), которые изучаются в качестве эффективной меры борьбы с ожирением и коморбидными состояниями, в том числе в сравнении с традиционным подходом, подразумевающим здоровое гипокалорийное питание. Проведенные исследования на животных моделях и испытания на людях показывают, что ПОВ может оказывать благотворное влияние на массу тела, его компонентный состав, сердечно-сосудистые параметры и, в ряде случаев, пищевое поведение. Тем не менее имеющиеся на сегодняшний день исследования отличаются малым объемом и значимой разнородностью выборок, небольшой продолжительностью наблюдений, сильно разнятся по используемым протоколам питания и длительности периодов голода/питания и в целом показывают неоднозначные результаты. В том числе нельзя отрицать тот факт, что положительные эффекты ПОВ могут быть опосредованы снижением калорийности рациона на фоне данных схем питания. Возможно, противоречивые результаты обусловлены тем, что метаболические эффекты ПОВ разнятся в зависимости от используемого окна питания в течение суток. Все больше данных свидетельствует о том, что для получения метаболических преимуществ прием пищи должен быть ограничен ранней фазой активности, т.е. утренним и дневным периодом времени. Однако широкое применение ранних схем интермиттирующего ограничения энергии может быть ограничено, в т.ч. в результате сложившихся в обществе социальных, семейных и производственных правил и традиций. Кроме того, не до конца понятно, как необходимость жесткого соблюдения предписанного окна питания будет влиять на пищевое поведение и психологическое здоровье граждан в долгосрочной перспективе.

Учитывая вышесказанное, можно заключить, что на сегодняшний день имеющиеся научные данные недостаточно убедительны, чтобы рекомендовать пациентам схемы ПОВ в качестве стандартной стратегии питания. Неизвестно, какие группы лиц получат наибольшую пользу от ПОВ и какая из его схем является наиболее эффективной. Возможно, ограниченное по времени питание принесет наибольшую пользу мотивированным людям, которые могут избежать гиперфагической реакции после периодов голодания и в то же время не связаны жесткими социальными и семейными обязательствами/традициями, ассоциированными с вечерними приемами пищи. Кроме того, решение следовать режимам ПОВ должно определяться индивидуальными целями и желаемыми результатами. И наконец, ПОВ может иметь потенциальные противопоказания, включая определенные состояния здоровья, прием лекарств, психосоциальные барьеры и пищевые привычки. Если ПОВ станет частью стандартной практики, его внедрение должно подразумевать использование междисциплинарного подхода. Взаимодействие сертифицированных диетологов, эндокринологов, кардиологов, гастроэнтерологов, фитнес-тренеров и других специалистов позволит обеспечить безопасность пациента и снизить вероятность побочных эффектов ПОВ, таких как повторный набор массы тела, депрессия, компульсивное переедание и другие нарушения пищевого поведения.

## ДОПОЛНИТЕЛЬНАЯ ИНФОРМАЦИЯ

Источники финансирования. Работа выполнена по инициативе авторов без привлечения финансирования.

Конфликт интересов. Авторы декларируют отсутствие явных и потенциальных конфликтов интересов, связанных с содержанием настоящей статьи.

Участие авторов. Берковская Марина Ароновна — получение, анализ данных или интерпретация результатов, написание статьи или внесение в рукопись существенной (важной) правки с целью повышения научной ценности статьи; Гурова Олеся Юрьевна — получение, анализ данных или интерпретация результатов, написание статьи или внесение в рукопись существенной (важной) правки с целью повышения научной ценности статьи; Хайкина Ирина Анатольевна — получение, анализ данных или интерпретация результатов, написание статьи или внесение в рукопись существенной (важной) правки с целью повышения научной ценности статьи; Фадеев Валентин Викторович — получение, анализ данных или интерпретация результатов, написание статьи или внесение в рукопись существенной (важной) правки с целью повышения научной ценности статьи. Все авторы одобрили финальную версию статьи перед публикацией, выразили согласие нести ответственность за все аспекты работы, подразумевающую надлежащее изучение и решение вопросов, связанных с точностью или добросовестностью любой части работы.

## References

[cit1] Vsemirnaya Organizatsiya Zdravookhraneniya. Tsentr SMI. Informatsionnyi byulleten' «Ozhirenie i izbytochnyi ves». Dostupno po: https://www.who.int/news-room/fact-sheets/detail/obesity-and-overweight. Ssylka aktivna na 26.07.2022.

[cit2] Yumuk Volkan, Tsigos Constantine, Fried Martin, Schindler Karin, Busetto Luca, Micic Dragan, Toplak Hermann (2015). European Guidelines for Obesity Management in Adults. Obesity Facts.

[cit3] Heymsfield Steven B, Harp Joyce B, Reitman Marc L, Beetsch Joel W, Schoeller Dale A, Erondu Ngozi, Pietrobelli Angelo (2018). Why do obese patients not lose more weight when treated with low-calorie diets? A mechanistic perspective. The American Journal of Clinical Nutrition.

[cit4] Pérez-Martínez P, Mikhailidis DP, Athyros VG, et al. Lifestyle recommendations for the prevention and management of metabolic syndrome: an international panel recommendation. Nutr Rev. 2017;75(5):307-326. doi: https://doi.org/10.1093/nutrit/nux014 28521334 PMC5914407

[cit5] Suliga Edyta, Kozieł Dorota, Cieśla Elżbieta, Rębak Dorota, Głuszek Stanisław (2017). Dietary Patterns in Relation to Metabolic Syndrome among Adults in Poland: A Cross-Sectional Study. Nutrients.

[cit6] Kant Ashima K., Graubard Barry I. (2014). 40-Year Trends in Meal and Snack Eating Behaviors of American Adults. Journal of the Academy of Nutrition and Dietetics.

[cit7] Pot Gerda K., Almoosawi Suzana, Stephen Alison M. (2016). Meal irregularity and cardiometabolic consequences: results from observational and intervention studies. Proceedings of the Nutrition Society.

[cit8] Ha Kyungho, Song YoonJu (2019). Associations of Meal Timing and Frequency with Obesity and Metabolic Syndrome among Korean Adults. Nutrients.

[cit9] Wilkinson Michael J., Manoogian Emily N.C., Zadourian Adena, Lo Hannah, Fakhouri Savannah, Shoghi Azarin, Wang Xinran, Fleischer Jason G., Navlakha Saket, Panda Satchidananda, Taub Pam R. (2019). Ten-Hour Time-Restricted Eating Reduces Weight, Blood Pressure, and Atherogenic Lipids in Patients with Metabolic Syndrome. Cell Metabolism.

[cit10] Xie Yanling, Tang Qingming, Chen Guangjin, Xie Mengru, Yu Shaoling, Zhao Jiajia, Chen Lili (2019). New Insights Into the Circadian Rhythm and Its Related Diseases. Frontiers in Physiology.

[cit11] Mason Ivy C., Qian Jingyi, Adler Gail K., Scheer Frank A. J. L. (2020). Impact of circadian disruption on glucose metabolism: implications for type 2 diabetes. Diabetologia.

[cit12] Stenvers DJ, Scheer FAJL, Schrauwen P, la Fleur SE, Kalsbeek A. Circadian clocks and insulin resistance. Nat Rev Endocrinol. 2019;15(2):75-89. doi: https://doi.org/10.1038/s41574-018-0122-1 30531917

[cit13] Chellappa Sarah L., Vujovic Nina, Williams Jonathan S., Scheer Frank A.J.L. (2019). Impact of Circadian Disruption on Cardiovascular Function and Disease. Trends in Endocrinology & Metabolism.

[cit14] Chow Lisa S., Manoogian Emily N. C., Alvear Alison, Fleischer Jason G., Thor Honoree, Dietsche Katrina, Wang Qi, Hodges James S., Esch Nicholas, Malaeb Samar, Harindhanavudhi Tasma, Nair K. Sreekumaran, Panda Satchidananda, Mashek Douglas G. (2020). Time‐Restricted Eating Effects on Body Composition and Metabolic Measures in Humans who are Overweight: A Feasibility Study. Obesity.

[cit15] Lopez-Minguez J., Gómez-Abellán P., Garaulet M. (2016). Circadian rhythms, food timing and obesity. Proceedings of the Nutrition Society.

[cit16] de Cabo Rafael, Mattson Mark P. (2019). Effects of Intermittent Fasting on Health, Aging, and Disease. New England Journal of Medicine.

[cit17] Dashti Hassan S, Scheer Frank A J L, Saxena Richa, Garaulet Marta (2018). Timing of Food Intake: Identifying Contributing Factors to Design Effective Interventions. Advances in Nutrition.

[cit18] Dong Tiffany A., Sandesara Pratik B., Dhindsa Devinder S., Mehta Anurag, Arneson Laura C., Dollar Allen L., Taub Pam R., Sperling Laurence S. (2020). Intermittent Fasting: A Heart Healthy Dietary Pattern?. The American Journal of Medicine.

[cit19] Regmi Prashant, Heilbronn Leonie K. (2020). Time-Restricted Eating: Benefits, Mechanisms, and Challenges in Translation. iScience.

[cit20] Charlot Anouk, Hutt Fanny, Sabatier Eugénie, Zoll Joffrey (2021). Beneficial Effects of Early Time-Restricted Feeding on Metabolic Diseases: Importance of Aligning Food Habits with the Circadian Clock. Nutrients.

[cit21] Davidson A. J., Poole A. S., Yamazaki S., Menaker M. (2003). Is the food-entrainable circadian oscillator in the digestive system?. Genes, Brain and Behavior.

[cit22] Shimizu Hatsumi, Hanzawa Fumiaki, Kim Daeun, Sun Shumin, Laurent Thomas, Umeki Miki, Ikeda Saiko, Mochizuki Satoshi, Oda Hiroaki (2018). Delayed first active-phase meal, a breakfast-skipping model, led to increased body weight and shifted the circadian oscillation of the hepatic clock and lipid metabolism-related genes in rats fed a high-fat diet. PLOS ONE.

[cit23] Hamaguchi Yutaro, Tahara Yu, Hitosugi Masashi, Shibata Shigenobu (2015). Impairment of Circadian Rhythms in Peripheral Clocks by Constant Light Is Partially Reversed by Scheduled Feeding or Exercise. Journal of Biological Rhythms.

[cit24] Kolbe Isa, Leinweber Brinja, Brandenburger Matthias, Oster Henrik (2019). Circadian clock network desynchrony promotes weight gain and alters glucose homeostasis in mice. Molecular Metabolism.

[cit25] Wang Hong, van Spyk Elyse, Liu Qiang, Geyfman Mikhail, Salmans Michael L., Kumar Vivek, Ihler Alexander, Li Ning, Takahashi Joseph S., Andersen Bogi (2017). Time-Restricted Feeding Shifts the Skin Circadian Clock and Alters UVB-Induced DNA Damage. Cell Reports.

[cit26] Gnocchi Davide, Bruscalupi Giovannella (2017). Circadian Rhythms and Hormonal Homeostasis: Pathophysiological Implications. Biology.

[cit27] Yu Haoyong, Xia Fuzhen, Lam Karen SL, Wang Yu, Bao Yuqian, Zhang Jialiang, Gu Yunjuan, Zhou Pengcheng, Lu Junxi, Jia Weiping, Xu Aimin (2011). Circadian Rhythm of Circulating Fibroblast Growth Factor 21 Is Related to Diurnal Changes in Fatty Acids in Humans. Clinical Chemistry.

[cit28] Fisher Ffolliott Martin, Maratos-Flier Eleftheria (2015). Understanding the Physiology of FGF21. Annual Review of Physiology.

[cit29] Gavrila Alina, Peng C.-K., Chan Jean L., Mietus Joseph E., Goldberger Ary L., Mantzoros Christos S. (2003). Diurnal and Ultradian Dynamics of Serum Adiponectin in Healthy Men: Comparison with Leptin, Circulating Soluble Leptin Receptor, and Cortisol Patterns. The Journal of Clinical Endocrinology & Metabolism.

[cit30] Gamble Karen L., Berry Ryan, Frank Stuart J., Young Martin E. (2014). Circadian clock control of endocrine factors. Nature Reviews Endocrinology.

[cit31] Barnea Maayan, Chapnik Nava, Genzer Yoni, Froy Oren (2014). The circadian clock machinery controls adiponectin expression. Molecular and Cellular Endocrinology.

[cit32] Yanai Hidekatsu, Yoshida Hiroshi (2019). Beneficial Effects of Adiponectin on Glucose and Lipid Metabolism and Atherosclerotic Progression: Mechanisms and Perspectives. International Journal of Molecular Sciences.

[cit33] Natalucci G, Riedl S, Gleiss A, Zidek T, Frisch H (2005). Spontaneous 24-h ghrelin secretion pattern in fasting subjects: maintenance of a meal-related pattern. European Journal of Endocrinology.

[cit34] Chan Jean L., Bullen John, Lee Jennifer H., Yiannakouris Nikos, Mantzoros Christos S. (2004). Ghrelin Levels Are Not Regulated by Recombinant Leptin Administration and/or Three Days of Fasting in Healthy Subjects. The Journal of Clinical Endocrinology & Metabolism.

[cit35] Moran-Ramos Sofía, Baez-Ruiz Adrian, Buijs Ruud M., Escobar Carolina (2016). When to eat? The influence of circadian rhythms on metabolic health: are animal studies providing the evidence?. Nutrition Research Reviews.

[cit36] Manoogian Emily N.C., Panda Satchidananda (2016). Circadian rhythms, time-restricted feeding, and healthy aging. Ageing Research Reviews.

[cit37] Yamauchi T, Kadowaki T (2009). Physiological and pathophysiological roles of adiponectin and adiponectin receptors in the integrated regulation of metabolic and cardiovascular diseases. International Journal of Obesity.

[cit38] D’souza AM, Neumann UH, Glavas MM, Kieffer TJ. The glucoregulatory actions of leptin. Mol Metab. 2017;6(9):1052-1065. doi: https://doi.org/10.1016/j.molmet.2017.04.011 28951828 PMC5605734

[cit39] Kolbe Isa, Brehm Niklas, Oster Henrik (2018). Interplay of central and peripheral circadian clocks in energy metabolism regulation. Journal of Neuroendocrinology.

[cit40] Albrecht Urs (2012). Timing to Perfection: The Biology of Central and Peripheral Circadian Clocks. Neuron.

[cit41] Njike Valentine Yanchou, Smith Teresa M, Shuval Omree, Shuval Kerem, Edshteyn Ingrid, Kalantari Vahid, Yaroch Amy L (2016). Snack Food, Satiety, and Weight. Advances in Nutrition.

[cit42] Yoshida Junko, Eguchi Eri, Nagaoka Kenjiro, Ito Tatsuo, Ogino Keiki (2018). Association of night eating habits with metabolic syndrome and its components: a longitudinal study. BMC Public Health.

[cit43] Vera Beatriz, Dashti Hassan S., Gómez-Abellán Purificación, Hernández-Martínez Antonio M., Esteban Alberto, Scheer Frank A. J. L., Saxena Richa, Garaulet Marta (2018). Modifiable lifestyle behaviors, but not a genetic risk score, associate with metabolic syndrome in evening chronotypes. Scientific Reports.

[cit44] Roßbach Sarah, Diederichs Tanja, Nöthlings Ute, Buyken Anette E, Alexy Ute (2017). Relevance of chronotype for eating patterns in adolescents. Chronobiology International.

[cit45] St-Onge Marie-Pierre, Ard Jamy, Baskin Monica L., Chiuve Stephanie E., Johnson Heather M., Kris-Etherton Penny, Varady Krista (2017). Meal Timing and Frequency: Implications for Cardiovascular Disease Prevention: A Scientific Statement From the American Heart Association. Circulation.

[cit46] Reutrakul Sirimon, Hood Megan M., Crowley Stephanie J., Morgan Mary K., Teodori Marsha, Knutson Kristen L. (2013). The Relationship Between Breakfast Skipping, Chronotype, and Glycemic Control in Type 2 Diabetes. Chronobiology International.

[cit47] Odegaard Andrew O., Jacobs David R., Steffen Lyn M., Van Horn Linda, Ludwig David S., Pereira Mark A. (2013). Breakfast Frequency and Development of Metabolic Risk. Diabetes Care.

[cit48] Witbracht Megan, Keim Nancy L., Forester Shavawn, Widaman Adrianne, Laugero Kevin (2014). Female breakfast skippers display a disrupted cortisol rhythm and elevated blood pressure. Physiology & Behavior.

[cit49] Sharma K, Shah K, Brahmbhatt P, Kandre Y (2018). Skipping breakfast and the risk of coronary artery disease. QJM: An International Journal of Medicine.

[cit50] Rynders Corey A., Thomas Elizabeth A., Zaman Adnin, Pan Zhaoxing, Catenacci Victoria A., Melanson Edward L. (2019). Effectiveness of Intermittent Fasting and Time-Restricted Feeding Compared to Continuous Energy Restriction for Weight Loss. Nutrients.

[cit51] Mattson Mark P., Longo Valter D., Harvie Michelle (2016). Impact of intermittent fasting on health and disease processes. Ageing Research Reviews.

[cit52] Anton Stephen D., Moehl Keelin, Donahoo William T., Marosi Krisztina, Lee Stephanie A., Mainous Arch G., Leeuwenburgh Christiaan, Mattson Mark P. (2017). Flipping the Metabolic Switch: Understanding and Applying the Health Benefits of Fasting. Obesity.

[cit53] Moon Shinje, Kang Jiseung, Kim Sang Hyun, Chung Hye Soo, Kim Yoon Jung, Yu Jae Myung, Cho Sung Tae, Oh Chang-Myung, Kim Tae (2020). Beneficial Effects of Time-Restricted Eating on Metabolic Diseases: A Systemic Review and Meta-Analysis. Nutrients.

[cit54] Chaix Amandine, Zarrinpar Amir, Miu Phuong, Panda Satchidananda (2014). Time-Restricted Feeding Is a Preventative and Therapeutic Intervention against Diverse Nutritional Challenges. Cell Metabolism.

[cit55] Delahaye Laura B., Bloomer Richard J., Butawan Matthew B., Wyman Jacqueline M., Hill Jessica L., Lee Harold W., Liu Andrew C., McAllan Liam, Han Joan C., van der Merwe Mariè (2018). Time-restricted feeding of a high-fat diet in male C57BL/6 mice reduces adiposity but does not protect against increased systemic inflammation. Applied Physiology, Nutrition, and Metabolism.

[cit56] Gill Shubhroz, Le Hiep D., Melkani Girish C., Panda Satchidananda (2015). Time-restricted feeding attenuates age-related cardiac decline in Drosophila. Science.

[cit57] Hatori Megumi, Vollmers Christopher, Zarrinpar Amir, DiTacchio Luciano, Bushong Eric A., Gill Shubhroz, Leblanc Mathias, Chaix Amandine, Joens Matthew, Fitzpatrick James A.J., Ellisman Mark H., Panda Satchidananda (2012). Time-Restricted Feeding without Reducing Caloric Intake Prevents Metabolic Diseases in Mice Fed a High-Fat Diet. Cell Metabolism.

[cit58] Olsen Magnus Kringstad, Choi Man Hung, Kulseng Bård, Zhao Chun-Mei, Chen Duan (2017). Time-restricted feeding on weekdays restricts weight gain: A study using rat models of high-fat diet-induced obesity. Physiology & Behavior.

[cit59] Sundaram Sneha, Yan Lin (2016). Time-restricted feeding reduces adiposity in mice fed a high-fat diet. Nutrition Research.

[cit60] Villanueva Jesús E., Livelo Christopher, Trujillo Adriana S., Chandran Sahaana, Woodworth Brendon, Andrade Leo, Le Hiep D., Manor Uri, Panda Satchidananda, Melkani Girish C. (2019). Time-restricted feeding restores muscle function in Drosophila models of obesity and circadian-rhythm disruption. Nature Communications.

[cit61] Woodie Lauren N., Luo Yuwen, Wayne Michael J., Graff Emily C., Ahmed Bulbul, O'Neill Ann Marie, Greene Michael W. (2017). Restricted feeding for 9 h in the active period partially abrogates the detrimental metabolic effects of a Western diet with liquid sugar consumption in mice. Metabolism.

[cit62] Sherman Hadas, Frumin Idan, Gutman Roee, Chapnik Nava, Lorentz Axel, Meylan Jenny, le Coutre Johannes, Froy Oren (2010). Long-term restricted feeding alters circadian expression and reduces the level of inflammatory and disease markers. Journal of Cellular and Molecular Medicine.

[cit63] Tsai Ju-Yun, Villegas-Montoya Carolina, Boland Brandon B., Blasier Zachary, Egbejimi Oluwaseun, Gonzalez Raquel, Kueht Michael, McElfresh Tracy A., Brewer Rachel A., Chandler Margaret P., Bray Molly S., Young Martin E. (2012). Influence of dark phase restricted high fat feeding on myocardial adaptation in mice. Journal of Molecular and Cellular Cardiology.

[cit64] Kentish Stephen J., Hatzinikolas George, Li Hui, Frisby Claudine L., Wittert Gary A., Page Amanda J. (2018). Time-Restricted Feeding Prevents Ablation of Diurnal Rhythms in Gastric Vagal Afferent Mechanosensitivity Observed in High-Fat Diet-Induced Obese Mice. The Journal of Neuroscience.

[cit65] Hu Dandan, Mao Yilei, Xu Gang, Liao Wenjun, Ren Jinjun, Yang Huayu, Yang Jun, Sun Lejia, Chen Hongyu, Wang Wenda, Wang Yanan, Sang Xinting, Lu Xin, Zhang Hongbing, Zhong Shouxian (2018). Time-restricted feeding causes irreversible metabolic disorders and gut microbiota shift in pediatric mice. Pediatric Research.

[cit66] Zarrinpar Amir, Chaix Amandine, Yooseph Shibu, Panda Satchidananda (2014). Diet and Feeding Pattern Affect the Diurnal Dynamics of the Gut Microbiome. Cell Metabolism.

[cit67] Chaix Amandine, Lin Terry, Le Hiep D., Chang Max W., Panda Satchidananda (2018). Time-Restricted Feeding Prevents Obesity and Metabolic Syndrome in Mice Lacking a Circadian Clock. Cell Metabolism.

[cit68] Duncan M.J., Smith J.T., Narbaiza J., Mueez F., Bustle L.B., Qureshi S., Fieseler C., Legan S.J. (2016). Restricting feeding to the active phase in middle-aged mice attenuates adverse metabolic effects of a high-fat diet. Physiology & Behavior.

[cit69] Sutton Elizabeth F., Beyl Robbie, Early Kate S., Cefalu William T., Ravussin Eric, Peterson Courtney M. (2018). Early Time-Restricted Feeding Improves Insulin Sensitivity, Blood Pressure, and Oxidative Stress Even without Weight Loss in Men with Prediabetes. Cell Metabolism.

[cit70] Anton Stephen D., Lee Stephanie A., Donahoo William T., McLaren Christian, Manini Todd, Leeuwenburgh Christiaan, Pahor Marco (2019). The Effects of Time Restricted Feeding on Overweight, Older Adults: A Pilot Study. Nutrients.

[cit71] Gabel Kelsey, Hoddy Kristin K., Haggerty Nicole, Song Jeehee, Kroeger Cynthia M., Trepanowski John F., Panda Satchidananda, Varady Krista A. (2018). Effects of 8-hour time restricted feeding on body weight and metabolic disease risk factors in obese adults: A pilot study. Nutrition and Healthy Aging.

[cit72] LeCheminant James D., Christenson Ed, Bailey Bruce W., Tucker Larry A. (2013). Restricting night-time eating reduces daily energy intake in healthy young men: a short-term cross-over study. British Journal of Nutrition.

[cit73] Moro Tatiana, Tinsley Grant, Bianco Antonino, Marcolin Giuseppe, Pacelli Quirico Francesco, Battaglia Giuseppe, Palma Antonio, Gentil Paulo, Neri Marco, Paoli Antonio (2016). Effects of eight weeks of time-restricted feeding (16/8) on basal metabolism, maximal strength, body composition, inflammation, and cardiovascular risk factors in resistance-trained males. Journal of Translational Medicine.

[cit74] Stote Kim S, Baer David J, Spears Karen, Paul David R, Harris G Keith, Rumpler William V, Strycula Pilar, Najjar Samer S, Ferrucci Luigi, Ingram Donald K, Longo Dan L, Mattson Mark P (2018). A controlled trial of reduced meal frequency without caloric restriction in healthy, normal-weight, middle-aged adults. The American Journal of Clinical Nutrition.

[cit75] Kesztyüs Dorothea, Cermak Petra, Gulich Markus, Kesztyüs Tibor (2019). Adherence to Time-Restricted Feeding and Impact on Abdominal Obesity in Primary Care Patients: Results of a Pilot Study in a Pre–Post Design. Nutrients.

[cit76] Hutchison Amy T., Regmi Prashant, Manoogian Emily N.C., Fleischer Jason G., Wittert Gary A., Panda Satchidananda, Heilbronn Leonie K. (2019). Time‐Restricted Feeding Improves Glucose Tolerance in Men at Risk for Type 2 Diabetes: A Randomized Crossover Trial. Obesity.

[cit77] Jamshed Humaira, Beyl Robbie, Della Manna Deborah, Yang Eddy, Ravussin Eric, Peterson Courtney (2019). Early Time-Restricted Feeding Improves 24-Hour Glucose Levels and Affects Markers of the Circadian Clock, Aging, and Autophagy in Humans. Nutrients.

[cit78] Allaf M, Elghazaly H, Mohamed OG, et al. Intermittent fasting for the prevention of cardiovascular disease. Cochrane Database of Systematic Reviews 2021;1. Art. No.: CD013496. doi: https://doi.org/10.1002/14651858.CD013496.pub2. PMC8092432 33512717

[cit79] Zeb Falak, Wu Xiaoyue, Chen Lijun, Fatima Sadia, Haq Ijaz-ul, Chen Aochang, Majeed Fatima, Feng Qing, Li Min (2020). Effect of time-restricted feeding on metabolic risk and circadian rhythm associated with gut microbiome in healthy males. British Journal of Nutrition.

[cit80] Cai Hua, Qin Yue-Lan, Shi Ze-Ya, Chen Jin-Hui, Zeng Min-Jie, Zhou Wei, Chen Ru-Qun, Chen Zhi-Yuan (2019). Effects of alternate-day fasting on body weight and dyslipidaemia in patients with non-alcoholic fatty liver disease: a randomised controlled trial. BMC Gastroenterology.

[cit81] Gasmi Maha, Sellami Maha, Denham Joshua, Padulo Johnny, Kuvacic Goran, Selmi Walid, Khalifa Riadh (2018). Time-restricted feeding influences immune responses without compromising muscle performance in older men. Nutrition.

[cit82] Tinsley Grant M, Moore M Lane, Graybeal Austin J, Paoli Antonio, Kim Youngdeok, Gonzales Joaquin U, Harry John R, VanDusseldorp Trisha A, Kennedy Devin N, Cruz Megan R (2019). Time-restricted feeding plus resistance training in active females: a randomized trial. The American Journal of Clinical Nutrition.

[cit83] Tinsley Grant M., Forsse Jeffrey S., Butler Natalie K., Paoli Antonio, Bane Annie A., La Bounty Paul M., Morgan Grant B., Grandjean Peter W. (2016). Time-restricted feeding in young men performing resistance training: A randomized controlled trial. European Journal of Sport Science.

[cit84] Singh R. B., Cornelissen Germaine, Mojto Viliam, Fatima Ghizal, Wichansawakun Sanit, Singh Mukta, Kartikey Kumar, Sharma J. P., Torshin V. I., Chibisov Sergey, Kharlitskaya Elena, Al-bawareed O. A. (2019). Effects of circadian restricted feeding on parameters of metabolic syndrome among healthy subjects. Chronobiology International.

[cit85] Antoni Rona, Robertson Tracey M., Robertson M. Denise, Johnston Jonathan D. (2018). A pilot feasibility study exploring the effects of a moderate time-restricted feeding intervention on energy intake, adiposity and metabolic physiology in free-living human subjects. Journal of Nutritional Science.

[cit86] Martens Christopher R., Rossman Matthew J., Mazzo Melissa R., Jankowski Lindsey R., Nagy Erzsebet E., Denman Blair A., Richey James J., Johnson Sarah A., Ziemba Brian P., Wang Yang, Peterson Courtney M., Chonchol Michel, Seals Douglas R. (2020). Short-term time-restricted feeding is safe and feasible in non-obese healthy midlife and older adults. GeroScience.

[cit87] McAllister Matthew J., Pigg Brandon L., Renteria Liliana I, Waldman Hunter S. (2019). Time-restricted feeding improves markers of cardiometabolic health in physically active college-age men: a 4-week randomized pre-post pilot study. Nutrition Research.

[cit88] Bi Huashan, Gan Yong, Yang Chen, Chen Yawen, Tong Xinyue, Lu Zuxun (2015). Breakfast skipping and the risk of type 2 diabetes: a meta-analysis of observational studies. Public Health Nutrition.

[cit89] Azami Yasushi, Funakoshi Mitsuhiko, Matsumoto Hisashi, Ikota Akemi, Ito Koichi, Okimoto Hisashi, Shimizu Nobuaki, Tsujimura Fumihiro, Fukuda Hiroshi, Miyagi Chozi, Osawa Sayaka, Osawa Ryo, Miura Jiro (2018). Long working hours and skipping breakfast concomitant with late evening meals are associated with suboptimal glycemic control among young male Japanese patients with type 2 diabetes. Journal of Diabetes Investigation.

[cit90] Nakajima Kei, Suwa Kaname (2015). Association of hyperglycemia in a general Japanese population with late-night-dinner eating alone, but not breakfast skipping alone. Journal of Diabetes & Metabolic Disorders.

[cit91] Okada Chika, Imano Hironori, Muraki Isao, Yamada Keiko, Iso Hiroyasu (2019). The Association of Having a Late Dinner or Bedtime Snack and Skipping Breakfast with Overweight in Japanese Women. Journal of Obesity.

[cit92] Jakubowicz Daniela, Barnea Maayan, Wainstein Julio, Froy Oren (2013). High Caloric intake at breakfast vs. dinner differentially influences weight loss of overweight and obese women. Obesity.

[cit93] Ravussin Eric, Beyl Robbie A., Poggiogalle Eleonora, Hsia Daniel S., Peterson Courtney M. (2019). Early Time‐Restricted Feeding Reduces Appetite and Increases Fat Oxidation But Does Not Affect Energy Expenditure in Humans. Obesity.

[cit94] Cienfuegos Sofia, Gabel Kelsey, Kalam Faiza, Ezpeleta Mark, Wiseman Eric, Pavlou Vasiliki, Lin Shuhao, Oliveira Manoela Lima, Varady Krista A. (2020). Effects of 4- and 6-h Time-Restricted Feeding on Weight and Cardiometabolic Health: A Randomized Controlled Trial in Adults with Obesity. Cell Metabolism.

[cit95] Lowe Dylan A., Wu Nancy, Rohdin-Bibby Linnea, Moore A. Holliston, Kelly Nisa, Liu Yong En, Philip Errol, Vittinghoff Eric, Heymsfield Steven B., Olgin Jeffrey E., Shepherd John A., Weiss Ethan J. (2020). Effects of Time-Restricted Eating on Weight Loss and Other Metabolic Parameters in Women and Men With Overweight and Obesity. JAMA Internal Medicine.

[cit96] Parr Evelyn B., Devlin Brooke L., Lim Karen H. C., Moresi Laura N. Z., Geils Claudia, Brennan Leah, Hawley John A. (2020). Time-Restricted Eating as a Nutrition Strategy for Individuals with Type 2 Diabetes: A Feasibility Study. Nutrients.

[cit97] Stockman Mary-Catherine, Thomas Dylan, Burke Jacquelyn, Apovian Caroline M. (2018). Intermittent Fasting: Is the Wait Worth the Weight?. Current Obesity Reports.

[cit98] Hoddy Kristin K, Kroeger Cynthia M, Trepanowski John F, Barnosky Adrienne R, Bhutani Surabhi, Varady Krista A (2015). Safety of alternate day fasting and effect on disordered eating behaviors. Nutrition Journal.

[cit99] Parr Evelyn B., Devlin Brooke L., Radford Bridget E., Hawley John A. (2020). A Delayed Morning and Earlier Evening Time-Restricted Feeding Protocol for Improving Glycemic Control and Dietary Adherence in Men with Overweight/Obesity: A Randomized Controlled Trial. Nutrients.

[cit100] Boivin D.B., Boudreau P. (2014). Impacts of shift work on sleep and circadian rhythms. Pathologie Biologie.

[cit101] Elgar Frank J., Craig Wendy, Trites Stephen J. (2012). Family Dinners, Communication, and Mental Health in Canadian Adolescents. Journal of Adolescent Health.

[cit102] Vesnaver Elisabeth, Keller Heather H. (2011). Social Influences and Eating Behavior in Later Life: A Review. Journal of Nutrition in Gerontology and Geriatrics.

[cit103] Kimura Yumi, Wada T., Okumiya K., Ishimoto Y., Fukutomi E., Kasahara Y., Chen W., Sakamoto R., Fujisawa M., Otsuka K., Matsubayashi Kozo (2012). Eating alone among community-dwelling Japanese elderly: Association with depression and food diversity. The journal of nutrition, health & aging.

[cit104] Carlson Olga, Martin Bronwen, Stote Kim S., Golden Erin, Maudsley Stuart, Najjar Samer S., Ferrucci Luigi, Ingram Donald K., Longo Dan L., Rumpler William V., Baer David J., Egan Josephine, Mattson Mark P. (2007). Impact of reduced meal frequency without caloric restriction on glucose regulation in healthy, normal-weight middle-aged men and women. Metabolism.

